# Report on the 23rd FEBS Young Scientists' Forum 2024

**DOI:** 10.1002/2211-5463.13933

**Published:** 2024-12-02

**Authors:** Riccardo Miggiano, Luigi Ippolito, Chiara Paganini, Alessio Paone, Francesca Tonelli, Sonia Trojan, Irene Díaz‐Moreno

**Affiliations:** ^1^ Department of Pharmaceutical Sciences University of Piemonte Orientale Novara Italy; ^2^ Department of Experimental and Clinical Biomedical Sciences “Mario Serio” University of Florence Italy; ^3^ Department of Research, Centre for Inherited Diseases Fondazione IRCCS Policlinico San Matteo Pavia Italy; ^4^ Department of Biochemical Sciences “A. Rossi Fanelli” Sapienza University of Rome Italy; ^5^ Biochemistry Unit, Department of Molecular Medicine University of Pavia Italy; ^6^ Chair of Medical Biochemistry, Faculty of Medicine Jagiellonian University – Medical College Krakow Poland; ^7^ Institute for Chemical Research (IIQ), Scientific Research Center “Isla de la Cartuja” (cicCartuja) University of Seville – CSIC Spain

**Keywords:** FEBS, meeting report, YSF

## Abstract

The 23rd FEBS YSF was held from 26th to 29th June 2024 in Pavia, Italy. Over 100 PhD students and early postdoctoral researchers from around 30 different countries came together at the inspiring rooms of the University of Pavia for a four‐day event. This year's topic was ‘Biochemistry for bridging the gap’, meaning the opportunity to have a comprehensive perspective on all biochemistry applications. Four renowned keynote speakers presented their latest research, accompanied by four career‐focused speakers, as well as additional sessions on academic career opportunities, including fellowships, women in science, and laboratory sustainability. Additionally, 10 selected YSF participants gave short talks to a large audience, while the remaining attendees shared their research findings through flash talks and two dedicated poster sessions. Scientific exchange and networking were encouraged during the poster sessions, breaks, and the social events. The meeting was a prelude before attending the 48th FEBS congress, celebrated in Milan. The success of the series will be continued during the 24th YSF edition: ‘Inspired by nature, driven by science’, which will take place from 2nd to 5th July 2025 in Sapanca, Türkiye.

**Abbreviations**
ENABLE: European Academy for Biomedical Science
FEBS: Federation of European Biochemical Societies
IUBMB: International Union of Biochemistry and Molecular Biology
SIB: Italian Society of Biochemistry and Molecular Biology
YSF: Young Scientists' Forum

Since its foundation, FEBS has prioritized organizing conferences to bring together professionals across disciplines in the broad field of biochemistry. The field itself has always thrived on the dedication of young scientists, who contributed to it with their hard work, experiments, collaboration, networking, and by exchanging ideas and discussions at both national and international meetings. The idea of gathering young biochemistry scientists from across Europe has been developed along the years through the settlement of the FEBS YSF.

The 23rd edition of the YSF took place in the city of Pavia, located in the northern region of Italy, from 26th to 29th June 2024. The meeting was hosted by the University of Pavia, one of the oldest universities in the world. The entire conference was organized by a group of six young and enthusiastic scientists, coordinated by several senior scientists from the hosting University as well as by members of the FEBS Careers of Young Scientists Committee (Table 1). The organizers took care of the scientific program, the social activities, and all the meeting logistics.

**Table 1:** Members of the YSF 2024 Organizing Committee and Committee Advisors.

**Organizing Committee**		
Riccardo Miggiano (chair)	University of Piemonte Orientale, Italy	Representative of Young Section of SIB
Luigi Ippolito	University of Florence, Italy	
Chiara Paganini	University of Pavia and Fondazione IRCCS Policlinico San Matteo Pavia, Italy	
Alessio Paone	Sapienza University of Rome, Italy	
Francesca Tonelli	University of Pavia, Italy	
Sonia Trojan	Jagiellonian University Medical College, Krakow, Poland	
**Committee Advisors**		
Antonella Forlino	University of Pavia, Italy	SIB representative
Vittorio Bellotti	University of Pavia, Italy	SIB representative
Irene Díaz‐Moreno	Institute for Chemical Research, University of Seville, Spain	FEBS Careers of Young Scientists Committee, Chair
Vlastimil Kulda	Charles University, Pilsen, Czech Republic	FEBS Careers of Young Scientists Committee
Anna Jagusiak	Jagiellonian University, Krakow, Poland	FEBS Careers of Young Scientists Committee

As alluded by the logo and the title of the forum ‘Biochemistry for bridging the gap’, the location took on a strictly metaphoric valency: the medieval bridge ‘Ponte coperto’ in Pavia was the symbol of what the organizers hoped for. That is, biochemistry as the way to connect different features from the molecular life sciences by an applicative point of view. The conference aimed to encourage young researchers to explore different perspectives within biochemistry by presenting their work in oral or poster sessions, discussing new experimental plans and cutting‐edge technologies, and being challenged by the emerging debates (mainly related to the biochemical basis of diseases, high‐throughput biomolecules identification for human exploitation, and sustainability).

To highlight such points, four keynote speakers were invited to present their work along the four‐day meeting. The speakers were selected for their impressive work in the respective biochemistry field and to bring a comprehensive idea of biochemistry to the meeting. Furthermore, 102 young scientists—PhD students and early career postdoctoral researchers—from 27 European countries were selected to participate in the event, in order to provide them with a unique opportunity present their research, and engage in constructive dialog with their peers and established experts in the field (Fig. 1). Of the 102 YSF awardees, 98 were supported by FEBS, which covered the accommodation during the YSF and the subsequent FEBS Congress, the registration fees for both events, and most of the travel expenses. Besides, two awardees were sponsored by the UK Biochemical Society and another two by the IUBMB, which provided financial support to two participants from Nigeria and Japan, making the conference more accessible to applicants from outside of Europe. Approximately 75% of the participants were female, with the majority being PhD students and postdocs (Fig. 2).

**Fig. 1:**
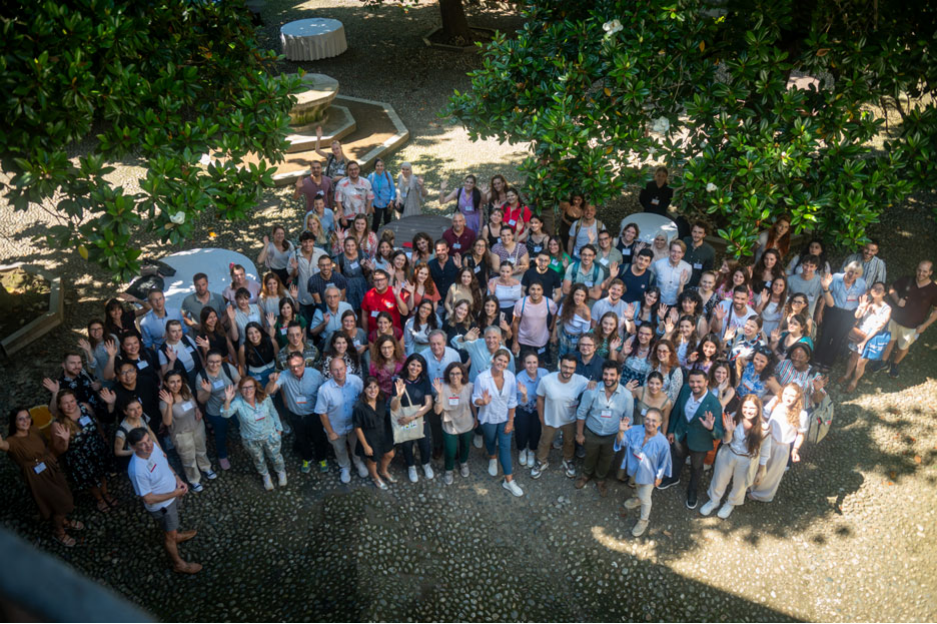
Group picture of the YSF 2024 participants in the courtyard of the University of Pavia.

**Fig. 2:**
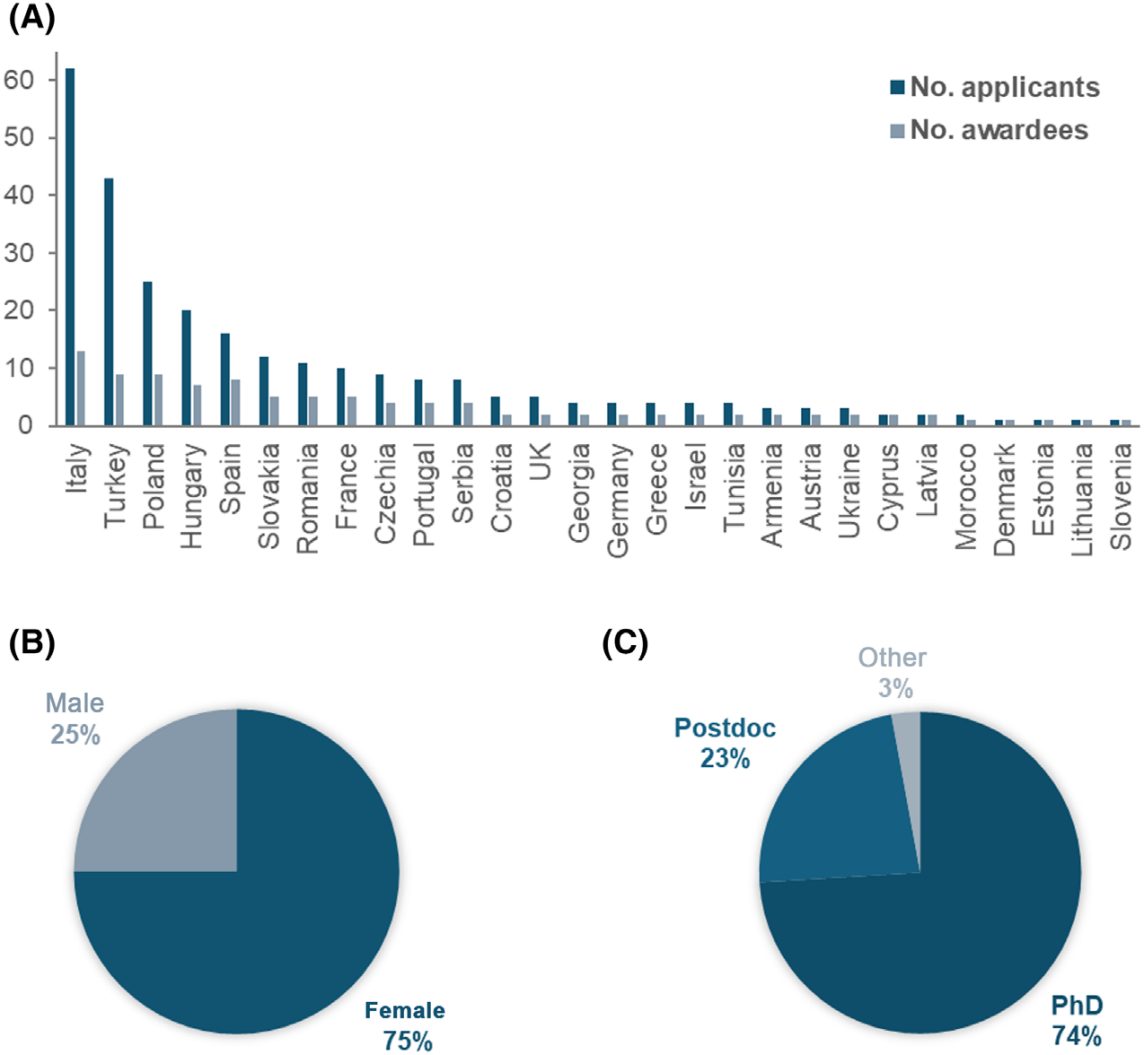
Overview of YSF 2024 participants. (A) Country distribution showing the number of YSF applicants and awardees of the FEBS‐funded YSF grants. (B) Gender distribution and (C) research profile of the YSF participants.

With an enthusiastic organizing team, more than 100 participants, excellent keynote and career speakers, and 4 days full of science and Italian fun, the YSF meeting in Pavia took place as follows.

## Scientific symposium

The scientific program of the event was conceived and constructed with the concept of biochemistry as a transversal discipline capable of revealing molecular details of interest to different, even seemingly unrelated, fields of research. Scientific seminars and presentations by participants selected for oral communication were held over 4 days, each with a specific theme. We invited four internationally renowned keynote speakers to present their research in different sessions spanning from molecular bases of human disease to the role of biochemistry in the circular economy to enhance sustainability.

The YSF started out with the official Opening Ceremony, where we formally welcomed the conference participants. Riccardo Miggiano, chair of the organizing committee, introduced the conference and said a few words about the story of the YSF and the purposes of the 2024 edition. The opening ceremony included speeches from the University of Pavia representatives Prof. Vittorio Bellotti and Prof. Antonella Forlino; the chair of FEBS Excellence Awards and Fellowships Committee, Prof. Alain Kroll, who presented the FEBS Excellence Awards meeting as a parallel event to the YSF 2024; the chair of FEBS Careers of Young Scientists Committee, Prof. Irene Díaz‐Moreno; and the FEBS Secretary General, Prof. Miguel Á. De la Rosa. The Major of the city of Pavia, Michele Lissia, also participated in the opening session.

**Fig. 3:**
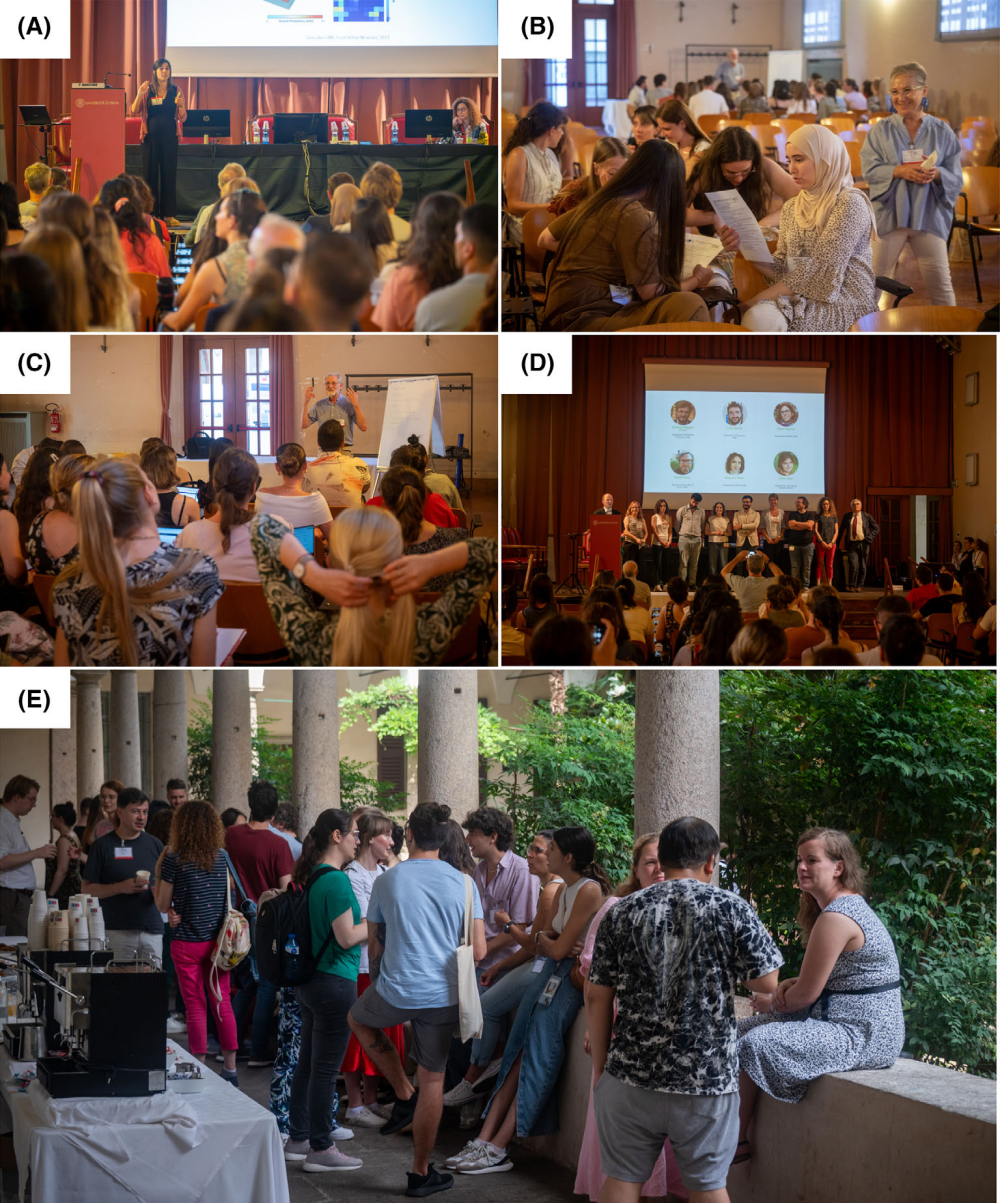
Some highlights from the YSF 2024 celebrated in Pavia. (A) Opening keynote lecture by Patrícia Monteiro. (B) Educator skills workshop delivered by Ferhan G. Sağın. (C) Career skills session on CV preparation presented by Keith Elliott. (D) Closing remarks with the members of the YSF organizing committee on the stage. (E) Relaxed chats and coffee break in the courtyard of the University of Pavia.

The YSF started with an opening lecture by Patrícia Monteiro (FEBS Excellence Awardee and group leader at the Faculty of Medicine of the University of Porto, Portugal). Patrícia presented her research in the context of synaptic and circuit mechanisms underlying autism spectrum disorder and illustrated how disruption of scaffolding proteins can affect brain circuitry and provide insightful links to neuropsychiatric disorders (Fig. 3A) [1]. In the evening, participants were invited to a welcome reception in the magnificent courtyard ‘Cortile dei Tassi’ of the University of Pavia.

The scientific program continued on the second day with a keynote lecture from Ana J. García‐Sáez (Professor at the Institute for Genetics, University of Cologne, Germany), who delivered a talk on the molecular mechanisms that govern membrane organization and cell death [2]. After Ana's lecture, the short talk awardees Ena Šimunić, Barbara Maria Contento, Karolina Gronkowska, H. Atakan Ekiz, and Tanakamol Mahawan highlighted their work in a 15‐min presentation. The afternoon included 1‐min flash talks by all those participants who presented their poster during the first poster session, as well as some career and social activities (see below).

The third day of the YSF started with a keynote lecture dedicated to structural biology by David Jeruzalmi (Professor of Chemistry and Biochemistry at The City College of New York, USA), who presented his structural studies on macromolecular complexes active in replicative helicase loading in bacteria [3]. As in the previous day, David's presentation was followed by the short talks of Marta Alberti, Edo Kiper, Marius Weismehl, Alicia L. Bruzos, and Ayelén Melina Santamans Recchini. These were proceeded by flash talks, the second poster session, and some more social activities.

The fourth and last day of the YSF was devoted to sustainability and environmental remediation. Marco Moracci (Professor of Biochemistry of the University of Naples ‘Federico II’ and associate researcher of the National Research Council of Italy) gave a keynote lecture on ‘Thermozymes for biorefineries: the biodiversity of extreme environments for the circular economy’ [4], which was followed by an online session dedicated to the Green Lab working initiative delivered by the FEBS Junior Section (see below). The scientific symposium ended with the presentation of the various activities supported by the FEBS Careers of Young Scientists Committee. Vlastimil Kulda (member of this Committee) presented the 3rd FEBS‐IUBMB‐ENABLE Conference, taking place in Singapore in December 2024; H. Atakan Ekiz and Duygu Harmancı, co‐chairs of the YSF 2025, presented the next edition of the YSF, taking place in Sapanca, Türkiye, in July 2025; and finally, Eszter Nagy‐Kanta, member of the FEBS Junior Section, presented the different activities for young scientists ran by the Junior Section.

Thanks to the generous support of FEBS and IUBMB journals, the following prizes were awarded: the *FEBS Open Bio* best oral communication prize was awarded to Marius Weismehl (Germany), the *FEBS Open Bio* poster prize to Patrícia Coelho (Portugal), and the IUBMB *BioFactors* poster prize to Malthe Kjær Bendtsten (Denmark). The meeting concluded with all the member of the organizing committee on stage, engaged in a collective embrace (Fig. 3D).

## Career skill workshops and social activities

The aim of the YSF was not only to foster scientific discussion, but young scientists also had the opportunity to engage directly with experts in various fields of research during the career skill sessions. During the second and third day of the YSF, participants were divided into four groups named after a famous Italian wine (Barolo from Piedmont, Falanghina from Campania, Negroamaro from Puglia, and Brunello di Montalcino from Tuscany). These career skill sessions included a workshop on curriculum vitae preparation delivered by Keith Elliott (member of the FEBS Education and Training Committee; UK) who provided tips on how to face the job market after the PhD (Fig. 3B); Jana Christopher (image data integrity analyst at FEBS Press; Germany) stressed the importance of publishing integrity, avoiding scientific misinterpretation and image manipulation; Ferhan G. Sağin (Chair of FEBS Education and Training Committee and Professor at Ege University Medical Faculty; Türkiye) underscore the significance of educator skills for young scientists (Fig. 3C); and Jason Perret (Professor emeritus of the Free University of Brussels Medical Faculty; Belgium) highlighted the importance of keeping an updated and comprehensive lab book. The career skill speakers offered a short presentation followed by practical exercises, also sharing their personal experience and providing helpful tips for everyday life in the lab.

In addition to the career skill workshops, the YSF program included a session on ‘Academic career opportunities’. Prof. Alain Krol, Chair of FEBS Excellence Awards and Fellowships Committee, presented the funding opportunities for young scientists supported by FEBS; Joanna Robaczewska and Edyta Niemczak, from the European Research Executive Agency, gave an online talk about the MSCA Postdoctoral Fellowships program; and Prof. Caroline Dean (Chair of the FEBS Women in Science Working Work) had a talk about equality encouraging all female scientists to lean in.

Two new activities were implemented in the 2024 edition of the YSF. A group of over 20 participants took part in the workshop ‘Creating an improvisational mindset’ delivered by the actor Thomas Chemnitz from the improvisational theater Berlin company ‘Die Gorillas’. Through fun practical and interactive exercises, the attendees explored how to enhance creativity, collaboration, and presentation skills through the art of improvisation. Besides, since the YSF was held in conjunction with the FEBS Excellence Awardees meeting, many YSF participants participated in informal mentoring chats with the Excellence awardees, junior PIs who provided first‐hand insights and advice on how to work towards an independent research position.

During the last day of the YSF, a ‘Green lab’ talk was delivered online by Patrick Penndorf, member of the FEBS Junior Section, raising awareness about sustainability in research. Sustainability and environmental remediation represented two key topics of the YSF 2024. Indeed, the YSF social program included a visit to ‘Centro Acqua & Sole’, an innovative company focused on circular economy directed by the heirs of the Nobel laureate Giulio Natta. This was followed by pleasant dinner in a typical Oltrepò pavese restaurant. In the perspective of stimulating social relations and networking between young scientists, a guided tour of Pavia was organized by the Erasmus Student network of Pavia, which gave the opportunity to YSF participants to admire the beautiful historic center of the town. A quick recap of some wonderful moments during the conference can be seen in Fig. 3.

## Conclusions

The 23rd FEBS Young Scientists' Forum in Pavia, Italy, focused on the theme of ‘Biochemistry for bridging the gap’, a concept that ran throughout the event. This idea went beyond metaphor, highlighting the interconnectedness of different levels of biological complexity, from atomic and molecular interactions to cellular and physiological processes. Young scientists were able to come together and discuss a broader view on life at all levels, extending beyond their own disciplines. A key success of this meeting was fostering interdisciplinary collaboration. Participants presented their work through short oral communication formats and poster sessions, creating a unique opportunity for researchers from diverse fields—such as structural biology or cell biology—to exchange experiences. This kind of discussion helped generate new ideas, thoughts, and approaches in a cooperative atmosphere. The selection of keynote speakers, recognized for their innovative work bridging again different areas of biochemistry, underscored the importance of a multi‐dimensional and integrated approach to scientific discovery.

In addition to the scientific discussion, YSF 2024 introduced mentorship activities, career skills workshops, and a focus on sustainability, encouraging participants to think beyond their research and consider its broader societal and environmental impacts. This holistic view echoed the wider aim of the forum, which was to advance biochemistry while ensuring that young scientists are equipped to navigate the increasingly interconnected world of modern science. The balanced mix of science, career seminars, and networking made the YSF 2024 a memorable and impactful event for its attendees. Looking ahead, the YSF 2025 in Sapanca, Türkiye, will continue this tradition, further breaking down barriers and encouraging new generations of cross‐discipline, global biochemists.

## Conflict of interest

The authors declare no conflict of interest.

## Author contributions

RM and IDM supervised the study and reviewed and edited the manuscript. LI, CP, AP, FT, and ST wrote the original draft of the manuscript.
